# Perspectives in systems nephrology

**DOI:** 10.1007/s00441-021-03470-3

**Published:** 2021-05-24

**Authors:** Maja T. Lindenmeyer, Fadhl Alakwaa, Michael Rose, Matthias Kretzler

**Affiliations:** 1 III. Department of Medicine, University Medical Center Hamburg-Eppendorf, Hamburg, Germany; 2 Department of Medicine, University of Michigan, Ann Arbor, MI USA

**Keywords:** System nephrology, Chronic kidney diseases, Precision medicine

## Abstract

Chronic kidney diseases (CKD) are a major health problem affecting approximately 10% of the world’s population and posing increasing challenges to the healthcare system. While CKD encompasses a broad spectrum of pathological processes and diverse etiologies, the classification of kidney disease is currently based on clinical findings or histopathological categorizations. This descriptive classification is agnostic towards the underlying disease mechanisms and has limited progress towards the ability to predict disease prognosis and treatment responses. To gain better insight into the complex and heterogeneous disease pathophysiology of CKD, a systems biology approach can be transformative. Rather than examining one factor or pathway at a time, as in the reductionist approach, with this strategy a broad spectrum of information is integrated, including comprehensive multi-omics data, clinical phenotypic information, and clinicopathological parameters. In recent years, rapid advances in mathematical, statistical, computational, and artificial intelligence methods enable the mapping of diverse big data sets. This holistic approach aims to identify the molecular basis of CKD subtypes as well as individual determinants of disease manifestation in a given patient. The emerging mechanism-based patient stratification and disease classification will lead to improved prognostic and predictive diagnostics and the discovery of novel molecular disease-specific therapies.

## Introduction

The kidney with its multiple specialized cell populations interacting in the complex three-dimensional nephron structure is responsible for maintaining the internal homeostasis of the human organism. Due to this central role, a dysregulation of kidney function can have a multitude of detrimental effects on the body. Kidney diseases are a major health problem with currently around 10% of the world’s population being affected by chronic kidney disease (CKD) (Collins and Foley 2012). They encompass a wide range of pathological processes and different etiologies which are a confluence of interactions of genetic, immune-mediated, environmental, and psycho-social factors. The classification of kidney diseases is currently based on clinical findings or histopathological categories. However, these clinical pathological classifications of kidney diseases lumping patients with similar phenotypes, but heterogenous underlying disease mechanisms resulting in inaccurate prediction of disease prognosis and treatment response. One reason for the slow progress in identifying and developing new therapies is the classic reductionist approach of the last decades, which examines one factor or one pathway at a time. While this approach has been successful in elucidating the physiological and pathophysiological function of specific genes or proteins in multiple monogenic kidney diseases, it has been less efficient in unraveling the complex interactions across genes, proteins, and pathways in diseases with complex disease pathogenesis. To overcome some of these challenges, a systems biology approach which integrates a wide spectrum of information including comprehensive multi-omics data, clinical phenotypic patient data, and clinicopathologic parameters, can complement the reductionist view and provide a more holistic understanding of the interacting disease mechanism in a given patient. In the last years, significant progress has been made in data modelling approaches and computational capabilities and methods to integrate diverse large sets of data. Application of different mathematical, statistical, and computational methods as well as the use of artificial intelligence data mining strategies can help to link molecular insight with clinically phenotypic data to better classify diseases, stratify patients, and design novel diagnostic and therapeutic tools.

The ultimate goal of the systems biology approach in medicine is to understand cellular function and interaction in a complex organ system and how perturbations relate to disease development and progression. The advancement of biomedical technologies able to comprehensively assess a specific molecular domain in human biosamples provides now datasets of sufficient depth to start to define cross cutting disease mechanism. Most notably we have seen rapid adaption of next-generation sequencing technology via RNA-seq, and scRNA-seq, and GC–MS via metabolomics and proteomics to collect genome scale information from human renal tissue obtained by biopsy. This is particularly impactful for renal research, as kidney cellular physiology will benefit greatly from the integration of different omics data across the different layers of data sets and nephron segment (Subramanian et al. 2020).

### Approaches

Integrating omics data can be subdivided into two main concepts: the post-analysis approach and the combination approach. The first approach integrates omics data by analyzing each omics data separately and then validating the results with other, orthogonal omics data. This approach is based on the integration of observations from different analyses of omics data (Pinu et al. 2019). There are two methods: top-down and bottom-up data reduction (Yu and Zeng 2018). The top-down method uses genomic and transcriptomic data to predict phenotypic responses and identify enriched signaling pathways, which are then validated by targeted metabolomics and proteomics. The disadvantage of this approach is that changes in genes, proteins, and metabolites do not necessarily directly correlate. In the bottom-up approach, significantly different metabolites are used to focus on the upstream pathways responsible for their alterations. The low coverage of metabolomics (one hundred measured metabolites versus thousands of measured genes and proteins) has been a major disadvantage of this approach, which limited the interpretation towards a comprehensive mapping across the multiscalar data domains to date.

In the combination approach, omics data is combined prior to data interoperation and visualization. The idea is to identify similarities between different omics data using mathematical methods such as Canonical Correlation Analysis (CCA) (Rohart et al. 2017) and orthogonal Two-Way Projection on Latent Structures (O2PLS) (Bouhaddani et al. 2016). More details on the methodologies and statistics behind these approaches can be found in the review from Subramanian (Subramanian et al. 2020).

## Which datasets can be used in systems nephrology

### Omics data

#### Genomics

Genome-wide association studies (GWAS) have become a valuable tool to decipher the polygenic architecture of complex diseases such as chronic kidney disease by identifying common genetic variants that are associated with complex diseases and traits. Despite the identification of thousands of disease- and/or trait-associated single-nucleotide polymorphisms (SNPs), the mechanism how these genetic variants impact gene regulation and the pathophysiological context is still largely unknown. One approach to investigate the influence of genetic variants on disease development is the integration of GWAS data with expression of downstream efforts, i.e., transcriptome data, so-called eQTL studies. In recent years, kidney compartment-based eQTL studies have started to provide context of disease-relevant processes and new targets as well as to establish kidney relevant eQTL databases. (NephQTL: http://nephqtl.org; Human kidney eQTL atlas: http://susztaklab.com/eqtl/) (Gillies et al. 2018; Qiu et al. 2018).

In order to obtain information regarding the whole spectrum of genetic mutations such as deletions, substitutions, and copy number variations, the technological development of whole exome sequencing (WES) or whole genome sequencing (WGS) can be deployed on kidney disease populations. These applications are already playing an important role in pediatric renal diseases, since about 70% of inherited kidney diseases are associated with childhood CKD (Gulati et al. 2020). Intriguingly also in adults about 10% of kidney diseases are due to a genetic cause (Groopman et al. 2019). Thus, these new technologies may lead to new insights in adult nephrology allowing a reclassification of diagnosis and treatment (Leveson and Oates 2020; Wilson et al. 2020).

#### Epigenomics

The advances in high-throughput sequencing technologies in recent years also facilitate a precise analysis of the epigenetic landscape. Approaches include among others the measurement of transcription factor (TF) binding and histone modifications by chromatin immunoprecipitation sequencing (ChIP-seq), and the detection of DNA methylation by bisulfite sequencing, the analysis of chromatin accessibility using the Assay of Transposase Accessible Chromatin Sequencing (ATAC-seq) (Li et al. 2019; Wilson et al. 2020).

DNA methylation represents an important epigenetic alteration that strongly influences gene expression and thereby playing an important role in regulating various physiological and pathological processes. In mammalians, DNA methylation occurs mainly at C5 of the cytosine ring within cytosine guanine (CpG) dinucleotides and is often found bundled in so called “CpG islands” at gene regulatory sites such as promoter regions. Several methods exist to study DNA methylation, with bisulfite sequencing being the gold standard for measuring the DNA methylation status of a genome. The analysis of the DNA methylation status can help us to extend our understanding of kidney development (Wanner et al. 2019) (and the pathophysiology of kidney diseases (Bansal et al. 2020).

Sequencing methods such as ATAC-seq (Assay of Transposase Accessible Chromatin) allow the systematic investigation of epigenetic mechanisms responsible for chromatin accessibility. In recent years single-cell/single-nucleus ATAC-Seq (scATAC/snATAC-seq) has been developed, which enables the analysis of cell type-specific chromatin accessibility in complex tissue with substantial cellular heterogeneity (Klemm et al. 2019). The identification of open chromatin in the genome at the single-cell level helps to refine our understanding of the functional heterogeneity and gene regulatory mechanisms in tissue such as the nephron (Muto et al. 2020).

#### Transcriptomics

##### RNA-seq/scRNA-seq

RNA sequencing is one of the next-generation sequencing technologies developed for the study of RNA expression, translation, and structure (Stark et al. 2019). Primary application and a routine research tool of RNA-seq is the identification of differential gene expression (DEGs), where we compare transcriptomic data from different samples and from different conditions. In bulk RNA-seq analysis, the average expression level for each gene present in the cell population is measured over a large number of input cells.

The kidney represents one of the most complex organs in the body, posing a major challenge in terms of resolving cellular heterogeneity. To better understand the complexity of the kidney, various approaches have long been pursued to identify cell-specific signals from complex signatures, including microdissection into individual compartments, enrichment of single-cell types, or computational methods such as in silico deconvolution (Cohen et al. 2002; Ju et al. 2013; Rinschen et al. 2018; Soutourina et al. 2005). With the ongoing development of new technologies such as single-cell RNA-seq (scRNA-seq) or single-nucleus RNA-seq (snRNA-seq), alternative approaches for measuring expression levels for each gene in individual cells become available to better resolve cellular heterogeneity and diversity in the kidney in an unbiased way. Multiple cellular dissociation protocols have been developed to obtain single-cell or single-nuclear profiles from complex human tissues with complimentary advantages of the different technologies towards comprehensive coverage of tissue resident cell types (Slyper et al. 2020; Wu et al. 2019). The identification of rare cell populations and definition of cellular heterogeneity can provide great insights into the pathogenesis of kidney diseases and kidney development (Abedini et al. 2021; Lake et al. 2019; Lindström et al. 2018; Subramanian et al. 2019; Wang et al. 2018; Wu et al. 2019, 2018a; Zheng et al. 2020). One problem with scRNA-seq protocols is that due to dissociation in single-cell suspensions, cells lose their spatial arrangement information. However, spatial arrangement within an organ plays an important role to understand cell–cell interactions and their functions in an organ. In recent years, promising approaches to spatially resolved transcriptomics have been developed that attempt to combine the information richness of single-cell technology with spatial resolution (Liao et al. 2021). Further developments are needed to achieve high spatial resolution combined with high throughput scRNA-Seq. These new approaches will improve our understanding of molecular mechanisms and cell–cell interactions in the kidney, aiding in the development of more accurate kidney disease classification, patient stratification, and novel therapeutic approaches*.*

##### CITE-seq

One of the innovative new tools for studying single-cell biology is the cellular indexing of transcriptomes and epitopes by sequencing (CITE-seq). CITE-Seq represents a multimodal assay that allows simultaneous transcriptomic and proteomic phenotyping at the single-cell level. Oligonucleotide-labeled antibodies are used to efficiently integrate cellular protein and transcriptome measurements (Stoeckius et al. 2017).

#### Proteomics

The qualitative and quantitative protein composition of a cell is influenced by various factors such as the expression level of the respective gene, posttranslational modifications, or environmental factors. High-throughput analysis of protein expression, protein modification, and protein–protein interactions can be investigated using mass spectrometry (MS)-based techniques such as tandem MS. There are several studies that have employed urine proteomics for the non-invasive early detection of kidney disease (Bellei et al. 2018). In the future, newly developed technologies such as spatial proteomics will allow us to obtain information of individual proteins on subcellular level and thus help us to better understand cell biology (Bottek et al. 2020; Lundberg and Borner 2019).

Since the protein concentration does not only depend on the mRNA abundance but is also regulated by posttranscriptional processes, frequently only a moderate correlation between transcript level and protein level is observed (Liu et al. 2016). Integration of multiple omics-platforms might help to overcome this problem and help to gain further insights into kidney biology. By combining deep proteome data with mRNA sequencing data from native mouse podocytes, a comprehensive and quantitative map of mammalian podocytes has recently been created (Rinschen et al. 2018). This multi-layered expression atlas not only allows the identification of characteristic features of podocytes but also opens the way to the discovery of new disease genes for human proteinuric kidney disease, potential drug targets, and the prioritization of these for follow-up studies by integrating further data from e.g. human studies.

#### Metabolomics/Lipidomics

The metabolome, i.e., the entirety of all metabolites and small molecules of a biological sample, is the product of the interaction of the genome with its environment. Currently, around 40 k primary and secondary human metabolites have been identified (Wishart et al. 2018). Using NMR-spectroscopic and MS-based technologies such as liquid chromatography (LC), gas chromatography (GC), or capillary electrophoresis (CE) mass spectrometry, hundreds to thousands of metabolites can be precisely measured simultaneously. Metabolomics provides insight into the mechanisms of kidney disease (Abbiss et al. 2019), the discovery of new therapeutic options (Rhee 2015), and the early detection of chronic kidney disease (CKD) (Chen et al. 2019; Grams et al. 2018). The next challenge in metabolomics is to develop a mechanistic understanding of metabolic processes in space with cellular and subcellular spatial resolution (Neumann et al. 2020).

The lipidome represents a subset of the “metabolome” that describes the lipid profile within a cell. In recent years, rapid developments in mass spectrometry and NMR spectrometry have stimulated research investigating the role of lipids in kidney disease (Afshinnia et al. 2018b; Avela and Siren 2020). Lipidomics can provide further insights into the mechanistic understanding of dyslipidemia in CKD patients. For example, different studies have shown that with progression of renal insufficiency elevation of saturated free fatty acids is observed, which is accompanied by a decreased efficiency of beta-oxidation (Afshinnia et al. 2018a; Rhee et al. 2010). Additionally, lipidomics can serve as a source for identifying potential biomarkers for disease progression as shown in type 2 diabetic kidney disease (Afshinnia et al. 2019) or in a subcohort of the Chronic Renal Insufficiency Cohort (CRIC), a longitudinal outcome study of patients with different CKD stages (Afshinnia et al. 2016).

## Large-scale biological data beyond omics

Multiple additional biomedical large-scale data sources for integrative biology are currently emerging. New imaging techniques and analysis methods based on deep digital spatial image capture linked with machine learning–driven analysis algorithms have revolutionized the information content and usefulness which was reserved so far to the qualitative image analysis by the human expert. For example, digitalized whole-slide images of kidney biopsies can be used to automatically extract descriptive and quantitative histopathological features, which in turn can be then linked with AI-driven pattern extraction to improve be diagnostic classifications, disease prognosis, and therapy response (for further information see (Becker et al. 2020). Quantitative analysis of cross-sectional imaging, in particular magnetic resonance imaging (MRI), positron emission tomography (PET), computed tomography (CT), and ultrasound (US), is increasingly proposed as an alternative source of biomarkers to inform chronic kidney disease (CKD) management (Gooding et al. 2020). In recent years, the interest is increasingly moving towards advanced imaging techniques that are sensitive to structural and functional tissue characteristics such as perfusion, oxygenation, blood flow, glomerular filtration, tubular flow, fibrosis, inflammation, metabolism, and tissue composition (Granda et al. 2018; Jiang et al. 2019). Additional utility derives from the fact that these characteristics can be measured separately for left and right kidney and for cortex and medulla and that they can characterize functional and structural heterogeneity within those areas.

**Fig. 1:**
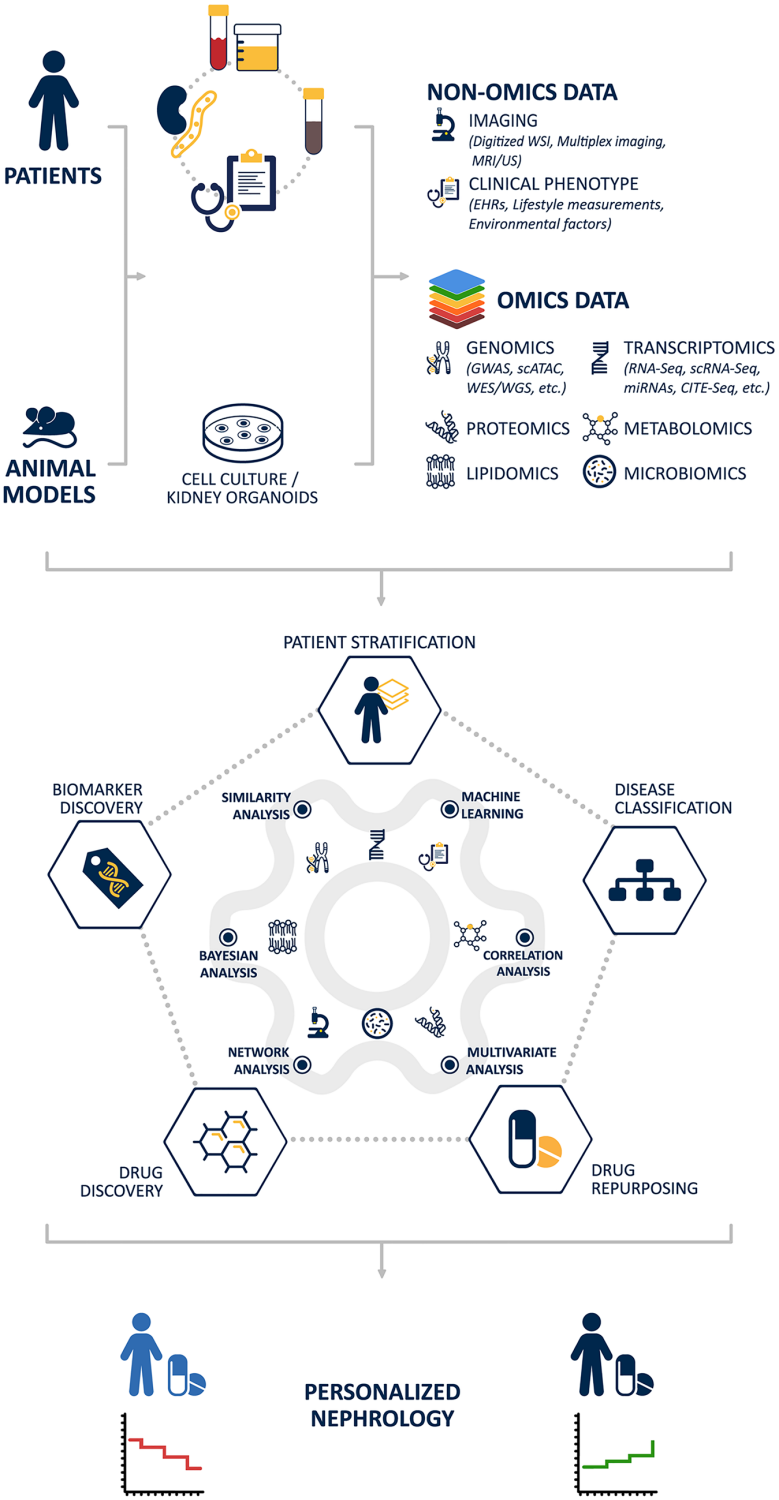
Schematic workflow of systems nephrology

Besides imaging data, clinical phenotypic data such as electronic health records, lifestyle data, or environmental exposures are an information-rich data source that can be incorporated into the systems biology workflow (Wu et al. 2017). In addition to electronic health records (EHRs) that contain information on the clinical profile of the patient such as diagnosis, biochemical parameters, or medication, lifestyle measurements such as physical activity, heart rate, sleep quality, or potassium level in sweat derived from health mobile applications (eHealth apps) as well as wearables have the potential to increase our understanding of disease epidemiology and help to detect patients at risk and improve patient compliance in disease management (Fig. 1).

## Data integration and network analysis

To gain a more holistic understanding of cellular function and interaction in the kidney and to learn how the phenotype of kidney disease is influenced by factors such as genes, proteins, or epigenetic factors, the integration of different levels of data is an important tool. Due to the complexity of the data, this poses a greater challenge. In recent years, however, various methods of data integration have been established which attempt to integrate specific subsets of omics data. This can be achieved using unsupervised and supervised approaches, including statistical or machine learning based methods, which can be further categorized into matrix factorization methods, correlation-based analysis, Bayesian methods, network-based methods, multiple kernel learning, and multi-step analysis. A complete survey of different methodologies is beyond the scope of this paper. A good overview of the different methodologies, limitations of the tools, and challenges of multi-omics data integration can be found in Huang et al. (2017) and Subramanian et al. (2020).

One of the data integration approaches involves network-based analysis. Biological networks aim to analyze different biological entities such as metabolites, genes, and proteins as an interacting system and its association with disease progression. There exist different types of biological networks such as protein–protein interaction (PPI), gene regulatory networks (GRN), signaling networks, neuronal networks, and metabolomics networks. Biological networks consist of two components, nodes and edges. While nodes can represent different biomolecules such as genes, proteins, and metabolites, edges represent the interaction of these bioentities. The type of interaction shown in the network depends on the definition of the interaction, i.e., for example, is it a physical interaction as in a PPI network or a regulation as in a GRN. Nodes with high connectivity represent a central gene, protein, or metabolite that serves as bridge between different portions of the network.

Many tools have been developed to create, visualize, and analyze biological networks. For example, correlation networks using WGCNA (Langfelder and Horvath 2008) and DiffCorr (Fukushima 2013) have been applied, e.g., to compare networks across health and disease stages and to evaluate how the connection of nodes is affected by disease state compared to healthy state. For scientists without programming skills, the web-based tool webCEMiTool https://cemitool.sysbio.tools/ offers a comprehensive modular analysis in a fully automated manner to perform WGCNA-based analysis of their data (Cardozo et al. 2019). In recent years, many user-friendly computational tools for scientists without a computational background have been build, which allow multi-omics data visualization, analysis and construction of correlation, gene regulatory, and PPI networks including Networkanalyst https://www.networkanalyst.ca/ (Zhou et al. 2019), RegNetwork http://www.regnetworkweb.org/home.jsp (Liu et al. 2015), OmicsNet http://www.omicsnet.ca (Zhou and Xia 2018), Mibiomics https://shiny-bird.univ-nantes.fr/app/Mibiomics (Zoppi et al. 2021), Paintomics http://www.paintomics.org/ (Hernandez-de-Diego et al. 2018), or Metaboanalyst https://www.metaboanalyst.ca/ (Chong et al. 2018). One of the best and very powerful network visualization and integration tool is Cytoscape https://cytoscape.org/ (Otasek et al. 2019; Shannon et al. 2003). Since its launch in 2003, more than 200 apps have been developed for complex network analysis and visualization. For further information on network-based analysis, refer to Ramos et al. (2019).

## Consortia

The comprehensive investigations of kidney disease patients using omics and non-omics data can hardly be accomplished by an individual research group or center but requires the establishment of complex infrastructures utilizing a distributed research networks of clinical centers, biobanks, registries, and highly specialized analytical laboratories, as is also done within the framework of the Collaborative Research Center described in this issue.

As potential molecular disease mechanism or identified biomarkers emerge a crucial part pertains to the validation in independent cohorts with different clinical, environmental, and genetic exposures.

In the past 20 years, many cohorts/consortia have been initiated to enable comprehensive studies of different kidney diseases and provide resources to the research community. Cohorts with relationship to the Collaborative Research Center are among others the ERCB (European Renal cDNA Bank), NEPTUNE (Nephrotic Syndrome Study Network), C-Probe (Clinical Phenotyping Resource and Biobank Core), the CRIC cohort (Chronic Renal Insufficiency Cohort), and the Kidney Precision Medicine Project (KPMP).

The ERCB has been launched more than 20 years ago and represents one of the first multi-center initiatives for comprehensive tissue level molecular analysis of human renal biopsies from patients with different chronic kidney diseases (Cohen et al. 2002). While the ERCB covers the entire renal disease spectrum observed in Europe, the NEPTUNE cohort study focuses mainly on patients with primary glomerular diseases (minimal change disease, focal segmental glomerulosclerosis, membranous nephropathy) (https://www.neptune-study.org/). NEPTUNE includes not only sampling of kidney biopsies but also urine and blood samples, collection of a wide range of demographic and clinical data, histopathological scoring and morphometry, whole genome sequencing, kidney compartment specific gene expression profiles, and comprehensive follow-ups of at least 36 months (Gadegbeku et al. 2013), with more than 800 patients enrolled. Two other cohorts of well-characterized patients with chronic kidney disease are C-PROBE (https://kidneycenter.med.umich.edu/clinical-phenotyping-resource-biobank-core) and the CRIC-study (http://www.cristudy.org/Chronic-Kidney-Disease/Chronic-Renal-Insufficiency-Cohort-Study/). C-PROBE is an ongoing, prospective multi-center cohort study of currently more than 1600 adult and pediatric participants with kidney disease from diverse clinical, ethnical, and socio-economic backgrounds. It encompasses a deep clinical phenotyping, as well as a collection of urine, blood, DNA, renal tissue biospecimen, and a longitudinal follow-up. The CRIC-study is one of the largest data and biospecimen collection in nephrology. To date, around 5.500 patients with different severity of CKD have been enrolled and characterized, more than 150.000 blood and urine biosamples as well as clinical data, data on quality of life, life style data and others, and longitudinal follow-ups have been collected. The KPMP project is a multidisciplinary effort by the nephrology research community with the goal to applying cutting-edge OMICs as well as imaging technologies on biopsy tissue obtained by research renal biopsies from AKI and CKD patients (https://www.kpmp.org/).

These data and samples serve as resources to the research community and help to pave the way to a more personalized medicine by defining disease subgroups, identifying cells, pathways, and targets for novel therapies.

## Application of systems biology approaches in Nephrology

An accurate disease taxonomy is important for the diagnosis and treatment of patients with CKD. At present, however, many patients with CKD often do not respond to treatment. The reason for this is, amongst others, the descriptive classification of diseases according to primarily presenting clinical symptoms and histopathological findings. Despite its comprehensive clinical application, this classification system does not reflect well individual patient factors and lacks the necessary understanding of the underlying molecular mechanisms required for effectively targeted treatment strategies. The employment of systems biology approaches can enable the identification of the underlying molecular disease mechanisms, individual patient factors, mechanism-based patient stratification, and disease classification as well as mechanism-based diagnostics using diagnostic and prognostic biomarkers and the discovery of new molecular, disease-specific therapies.

### Disease mechanism and disease classification

One goal of systems medicine is the identification of molecular disease mechanisms, which in turn enables the reclassification of diseases based on the underlying molecular mechanism and patient stratification based on their molecular characteristics. Linking molecular programs to structure and function on a cellular level has become an important tool not only for identifying and classifying cell types but also for uncovering disease mechanisms and refining disease classifications.

A recent study of the Kidney Precision Medicine Project (KPMP) (www.kpmp.org) can be used to illustrate the power of these approaches. The study used a deconvolution strategy by integrating scRNA data from human reference biopsy samples with bulk RNA seq data from patients with various chronic kidney diseases to identify cell type–specific gene signatures and define molecular subgroups in glomerular diseases (Kammer et al. 2019). Different reference tissue sources were used to create a reference atlas of 31 different renal cell types, including three different endothelial cell clusters. The scRNAseq-directed endothelial cell gene signatures enabled the generation of a glomerular endothelial cell (GEC) score, which after integration with CKD bulk RNA data led to the identification of two distinct groups of FSGS patients and showed an association between GEC activation and exposure to immunosuppressive treatment at time of biopsy. The analysis of the molecular endothelial gene signatures of the two FSGS subgroups revealed significant differences in intrarenal α-2-macroglobulin (A2M) gene expression levels and an association of A2M transcript levels with disease progression, suggesting A2M as a cell type-specific outcome predictor.

In studies published in 2019 (Arazi et al. 2019; Der et al. 2019), single-cell RNA-Seq technique has been applied to explore the heterogeneity of lupus nephritis (LN), decipher intercellular interactions, and identify novel prognostic markers. For this purpose, kidney and skin biopsy samples from patients with LN and healthy controls were analyzed. The scRNA-Seq analyses indicated that lupus patients experienced a higher IFN response in renal tubule cells and keratinocytes compared to healthy controls and that these correlated strongly with each other. Furthermore, they could show that specific molecular signatures of tubular epithelial cells and keratinocytes such as the upregulation of type I IFN-response and TNF signaling differentiated patients with proliferative LN from membranous or mixed LN. Besides the dysregulation of immune-related pathways, patients with lupus nephritis exhibited an upregulation of fibrosis markers in tubular epithelial cells and keratinocytes which also correlated with each other in the individual patients. Based on these results, Der and colleagues were able to establish IFN-response and fibrosis signatures in tubular epithelial cells, which allowed a prediction of treatment response 6 months after biopsy and might be used as potential prognostic biomarkers and stratification tools in the future. Finally, single-cell analysis of matching urine samples allowed the definition of a macrophage subtype present in both kidney tissue and urine with the potential for effective non-invasive monitoring of the intrarenal states.

Another interesting and powerful tool that has been developed in recent years for the identification and analysis of disease mechanisms are kidney organoids derived from human-induced pluripotent stem cells (iPSCs). By applying single-cell RNASeq technologies to kidney organoids, recent studies have shown on the one hand that the reproducibility and quality of kidney organoids derived from different human iPSC lines can be reliably assessed with scRNA-Seq (Subramanian et al. 2019). Secondly, data integration analysis of different single-cell data sets demonstrated the conservation of different cell types between kidney organoids and fetal kidney and provided evidence that kidney organoids can mimic normal fetal development in terms of cellular identity and complexity (Combes et al. 2019). In another study, a combination of single-cell data sets and bulk RNA-Seq data not only identified robust and reproducible gene expression signatures of cells present in organoid cultures shared with developing human kidneys but also detected a gene expression signature characteristic of developing glomerular epithelial cells in glomerular tissue of patients with CKD (Harder et al. 2019).

### Biomarker discovery

In addition to elucidating molecular mechanisms and reclassifying diseases, systems biology plays an important role in the discovery and development of diagnostic and prognostic biomarkers. Prediction of renal function loss is still difficult to achieve, as until now there are no reasonable biomarkers available in routine diagnostics that could improve the predictive power beyond the established markers ofc proteinuria and eGFR. In recent years, however, systems biology approaches have been used to identify prognostic markers for the differentiation of courses in CKD patients.

A recently published study explored the potential of multi-omics-derived biomarkers to improve the prediction of disease courses in patients with type 2 diabetes and incident or early CKD in addition to existing clinical predictors (Kammer et al. 2019). Applying Bayesian multi-variable logistic regression models, the authors analyzed 402 potential biomarker candidates, including clinical parameters, proteome, lipidome, and metabolome panel data, for their prognostic potential to distinguish eGFR trajectories in a cohort of patients with a stable eGFR course and with a rapid eGFR decline. Of these candidates, only KIM-1 and NTproBNP together with baseline eGFR contributed to a refined, but modest differentiation between stable and progressive courses, while the metabolomic and lipidomic biomarkers seemed to have no impact on the prognostic capability.

In recent years, several studies have suggested an influence of inflammation and inflammation-associated factors on DKD progression. For instance, the circulating TNFR family members TNFR1 and 2 have been shown to be promising predictive biomarkers for DKD progression in patients with type 1 and type 2 diabetes (Gohda et al. 2012; Niewczas et al. 2012). Using a customized SOMAscan platform of 194 inflammatory proteins, Niewczas and colleagues recently succeeded in identifying a kidney risk inflammatory signature (KRIS) consisting of 17 proteins associated with the development of ESRD (Niewczas et al. 2019). Among these 17 proteins, 6 were members of the TNFR superfamily, including TNFR1 and 2, serving as predictive biomarkers for renal outcome in patients with type 1 and type 2 diabetes.

Another example represents urinary EGF, which was identified as a prognostic marker for CKD progression through a systems biology approach based on kidney biopsy transcriptome data, urinary proteome data, and clinical follow-up data from CKD patients with different disease entities (Ju et al. 2015). Ju et al. did not only show that urinary EGF protein correlated positively with intrarenal EGF mRNA, which is mainly expressed in the distal tubules, but could also predict the risk of disease progression as demonstrated by the positive correlation of EGF with eGFR slope as a measure of loss of renal function. The integrative use of urinary EGF with the standard parameters proteinuria or eGFR could enhance their predictive power of disease outcome. Further studies have validated and confirmed the potential of urinary EGF as a prognostic marker for loss of renal function in a wide range of CKD patients with different disease entities (Azukaitis et al. 2019; Li et al. 2018; Segarra-Medrano et al. 2017; Wu et al. 2018b).

### Drug target discovery and drug repurposing

A key goal of systems medicine is the discovery of new therapeutic targets. To improve the process of target identification and drug development novel strategies are currently emerging. This includes systems-biology-based target discovery, drug repurposing, a process which attempts to identify new targets for already approved or investigational drugs as well as novel model systems like kidney organoids as drug screening platforms.

A good example of systems biology-based drug target discovery is the identification of JAK-STAT as a potential drug target for diabetic kidney disease (DKD). Using cross-species transcriptome analysis, Hodgin et al. demonstrated a key role for the JAK-STAT pathway in diabetic kidney disease (Hodgin et al. 2013). The causal role of the pathway was supported by podocyte-specific overexpression of JAK2 and treatment of mice with an oral JAK inhibitor (Zhang et al. 2017), ultimately leading to a phase 2 clinical trial in diabetic kidney disease. Treatment with the selective JAK1 and JAK2 inhibitor baricitinib resulted in a dose-dependent decrease in albuminuria, indicating a potential benefit of JAK1/JAK2 inhibitors as a new therapy for DKD patients (Tuttle et al. 2018). Another study in patients with focal segmental glomerulosclerosis (FSGS) demonstrated activation of the JAK/STAT pathway as a marker of renal disease progression, suggesting this pathway might be affected by drug treatment in patients with FSGS (Tao et al. 2018).

An attractive strategy to identify novel therapies for kidney diseases is drug repurposing, which can help reducing risks, costs, and time in drug development. In drug repurposing, both computational and experimental approaches can be employed. Computational approaches can include both omics data (such as transcriptome, genomic, or proteome data) as well as non-omics data of any kind (e.g., chemical structure or electronic health records (EHRs)). For the systematic analysis of the data, different approaches such as signature matching, pathway mapping, or genetic association can be applied individually or in combination (Pushpakom et al. 2019). For example, by using a transcriptome-based signature matching approach, Williams and colleagues were able to identify lysine deacetylase inhibition as a potential new treatment option for progressive CKD (Williams et al. 2020). In a first step, a chronic renal disease progression signature was defined using Col4a3-/- mice, which showed proteinuria and progressive loss of renal function. A comparison of this signature with the molecular signatures in the Connectivity Map database identified vorinostat, a lysine deacetylase inhibitor, as a candidate with potential impact on CKD progression. Treatment of Col4a3-/- mice with vorinostat was shown to significantly prolong the lifespan of the animals and exert renoprotective effects, indicating lysine deacetylase inhibition as potential treatment approach for chronic kidney disease. Previous studies in diabetic mice could already demonstrate renoprotective effects of vorinostat. The drug not only reduced albuminuria, mesangial collagen IV deposition, and oxidative-nitrosative stress in streptozotocin (STZ)-treated mice (Advani et al. 2011) but also revealed an inhibitory effect on diabetes-associated renal growth in STZ-treated rats, partly due to modulation of the EGF-EGFR axis (Gilbert et al. 2011), further supporting the potential of vorinostat as a treatment strategy for chronic kidney disease and the important role of the EGF pathway in CKD.

### Defining pattern in large data sets via artificial intelligence

The accumulation of large omics and non-omics data over the last two decades, advances in computer performance and the development of algorithms for deep and machine learning have fostered many applications of AI to develop data-driven early detection, diagnosis, and management of kidney disease (PD) (Yuan et al. 2020). One of the most recent AI applications in KD is a deep learning program developed by Google called “Deepmind” that can predict acute kidney injury (AKI) based on patient electronic health records (EHR) (Powles and Hodson 2017). Deepmind trained and tested on 703,782 adult patients and 620,000 characteristics in over 1243 healthcare facilities (sites) in the UK and the USA. The model was able to predict AKI episodes of hospitalized patients with a sensitivity of 55.8% up to 48 h in advance and a false alarm rate of 2:1, allowing physicians to intervene early enough to prevent patients' kidney failure. Although Deepmind is still in need of improvement in terms of its accuracy and further validation on other independent and more comprehensive population data sets (e.g., only 6% of the patients studied were female in the Deepmind training set), it opens the door for the incorporation of artificial intelligence into the clinical setting and could represent a potential approach to risk prediction for AKI in the future.

Another application of AI in non-omic data is the development of an automated computerized pipeline for annotation and classification of human kidney biopsies from digitized histological images. Recent studies demonstrated the successful use of convolutional neural networks (CNN) to automatically segment and classify transplant biopsies (Hermsen et al. 2019), biopsies of patients with diabetic kidney disease (Ginley et al. 2019), and the automated interpretation of immunofluorescence specimen of kidney biopsies (Ligabue et al. 2020). By applying deep-learning algorithms on whole slide images (WSI), Hermsen and colleagues achieved a multiclass segmentation of renal tissue in routinely PAS-stained sections. The algorithm displayed a robust performance in terms of sample preparation, scanning performance, and inter-laboratory differences and was able to successfully analyze both healthy and pathological tissue samples. In addition, significant concordance was achieved in the quantification of CNN segmentation data and the components of the Banff classification system visually assessed by renal pathologists in whole transplant biopsies (Hermsen et al. 2019). In the second study by Ginley et al., the authors successfully extracted and segmented glomerular boundaries, nuclei, and glomerular structures from whole slide images (WSIs) of human and murine diabetic kidney tissue using an iterative whole-slide CNN training interface—the human-artificial-intelligence-loop (HAIL). Despite the small sample size used, the classification approach showed a high sensitivity and specificity of the newly developed method and a moderate Cohen's Kappa *k* = 0.55 compared to senior pathologists, which is similar to a comparison among pathologists. Due to the flexibility of the pipeline, this work could be extended to other diseases such as IgA nephropathy or lupus nephritis and used for outcome prediction of numeric labels such as proteinuria. In addition to analyses of PAS images, immunofluorescence staining plays an important role in the histopathological evaluation of a disease (Ginley et al. 2019). The recent study by Ligabue and colleagues represents a first attempt to use AI-based methods in the evaluation and classification of IF-detected immune deposits in kidney biopsies. By analyzing 12,259 immunofluorescence images from 2542 kidney biopsies taken over the last 18 years, they were able to build an automated reporting pipeline of the key characteristics normally collected in immunofluorescence analysis of kidney biopsies using conventional neural networks. This was accomplished with a significant accuracy and comparable performance to visual inspection by human experts (Ligabue et al. 2020).

These studies show that AI can be a useful tool to improve research of kidney diseases and support clinical practice. However, till these methods can be introduced into routine diagnostics and clinical practice, further validations and improvements in algorithms are needed.

## Conclusion and outlook

As described in this review, emerging technologies and rapidly developing computer methods are opening up new horizons in nephrology. In this context, data integration of different data layers (omics and non-omics data) plays a crucial role.

However, despite major efforts to collect more accessible multi- and non-comics data, data integration still has many challenges (Subramanian et al. 2020). One of these challenges is the variety of protocols used to collect and store omics data, which are only suitable for individual types of omics. Therefore, minimum standards such as the already established quality standards for microarray (MIAME), RNA-Seq (MINSEQ), or proteome (MIAPE) experiments should generally be agreed upon for the design of experiments (data preparation and extraction) in order to allow data comparison between different omics types. This would lead to a general improvement in the quality of research when integrating omics data. On the other hand, the transparency and reproducibility of multi-omics data is a critical point. For example, most omics studies require samples to be stored at −80 °C or below and a fast processing time to prevent degradation of RNA, proteins, and metabolites. However, some omics experiments, such as metabolomics, are more sensitive to environmental disturbances such as temperature and humidity than other omics experiments such as proteomics, genomics, and transcriptomics. These interfering factors should be reported, documented, and published for each sample so that these factors can be taken into account by other researchers. Third, many omics data such as metabolomics, proteomics, and transcriptomics are poorly reproducible when produced on different platforms and in different laboratories, which limits the generalization of results. The use of reference standards, standardized protocols for sample storage and preparation, and quality control samples can improve the reproducibility of studies with omics data, while other factors such as the inherent bias of sampling are difficult to avoid. For an example of a comprehensive capture of omics related experimental data sets with a focus on experimental metadata see (El-Achkar et al. 2020).

In addition to these challenges, there are other factors that play a critical role and should be considered when integrating data. For example, the relationship from gene to protein to metabolite is not necessarily linearly proportional. Therefore, the correlation is not always associated with functional differences. Secondly, poorly designed multi-omics studies lead to false positive and negative results; i.e., the quality of the individual omics data must be checked and validated before integrating the various omics data. Furthermore, the number of samples required to extract meaningful results should be calculated for each omics study, because each individual omics data requires a different sample size; for example, untargeted metabolomics and proteomics studies (non-quantitative experiments) require a larger sample size than targeted experiments (quantitative experiments).

The recent efforts by many international projects such as KPMP (de Boer et al.  2021; Hansen et al. 2020) and HuBMAP (Hu 2019) try to overcome these challenges by providing access to large number of patients, standard operating procedures for sample and data collection, and data analysis. While with the current scRNA-seq and snRNA-SEQ omics technologies the mRNA expression from thousands of cells can be measured, the spatial locations of these cells are lost due to the required cell dissociation (Wilbrey-Clark et al. 2020). As the cell location in the tissue is important to understand its function, therefore, new technologies such as spatial transcriptomics (Lindström et al. 2020) which combines transcriptomics with imaging techniques have developed that help us putting cells into tissue context. The technology of spatial transcriptomics is still in a maturing phase, requires expensive imaging equipment, and is time- and labor-intensive and difficult to interpret in complex tissue such as the kidney. Meanwhile, many other tools have been developed to reconstruct 3D organs from RNA and proteins, such as CLARITY (Du et al. 2018) in the mouse and 3DISCO (Zhao et al. 2020), the first example in which an entire human brain and kidney were reconstructed in 3D using tissue clearing and deep learning methods. This in turn opens up new ways to better understand the molecular and structural architecture of organs. Other omics data technology such as spatial metabolomics (Neumann et al. 2020) have emerged providing further insights in a cell state.

In the near future, these new technologies and integrative analytical methods will contribute to a better understanding of the molecular pathophysiology kidney disease, molecular disease classification, and mechanistic patient stratification for clinical studies. This in turn will lead to improved diagnostics and to selecting the right treatment for each patient. Precision nephrology is moving ever closer.
